# Giant Cell Tumor of the Calcaneus

**DOI:** 10.7759/cureus.7467

**Published:** 2020-03-30

**Authors:** Dheeraj Batheja, Apoorv Sehgal, Avijeet Prasad, Pratyush Shahi, Kuldeep Bansal

**Affiliations:** 1 Orthopaedics, University College of Medicine Science and Guru Teg Bahadur Hospital, Delhi, IND; 2 Orthopaedics, University College of Medical Sciences and Guru Teg Bahadur Hospital, Delhi, IND

**Keywords:** giant cell tumor, calcaneus, gct, curettage, bone cementing, recurrence, calcaneum

## Abstract

A 17-year-old female presented to us with pain and swelling in the right heel. Examination revealed the swelling to be tender, hard and fixed to the calcaneus. Radiographs showed an expansile, lytic lesion of the calcaneus with well-defined margins and no extraosseus spread. A core biopsy was done which showed multinucleated giant cells in a sea of mononuclear stromal cells, suggestive of a giant cell tumour (GCT). Curettage and filling up of the defect with bone cement was done under anaesthesia. The patient was fully ambulatory three months after the surgery. At two-year follow-up, the patient continued to be asymptomatic and radiographs revealed no signs of recurrence. It is important to note that GCT can occur in these rare sites and unusual age groups, and hence requires a good level of awareness of the surgeon and adequate preoperative workup, including biopsy, before proceeding to the definitive treatment of the lesion. Considering its potential local aggressiveness, early intervention is necessary. The patient should be kept under regular follow-up to detect any recurrence or metastasis in early stage.

## Introduction

Giant cell tumour (GCT) of the calcaneus is a rare entity. In studies performed by Campanacci and Dahlin on bone GCTs (327 and 195 cases, respectively), incidence of calcaneal GCT was found to be less than 1% [1,2]. It is generally seen in the age group of 30-40 years and shows high recurrence rate and potentially aggressive features [3,4].

We report the case of a 17-year-old female with calcaneal GCT to increase awareness among clinicians about the existence of intraosseous GCTs in these rare sites and unusual age groups and necessity of early intervention in such cases to prevent complications.

## Case presentation

A 17-year-old female presented with right heel pain associated with swelling. The pain had been present for 11 months. However, the swelling had appeared six months previously. There was no history of trauma or fall. On examination, there was a prominent swelling at the right heel which was tender, hard and fixed to the calcaneus. The range of motion of the ankle joint was normal. 

**Figure 1:**
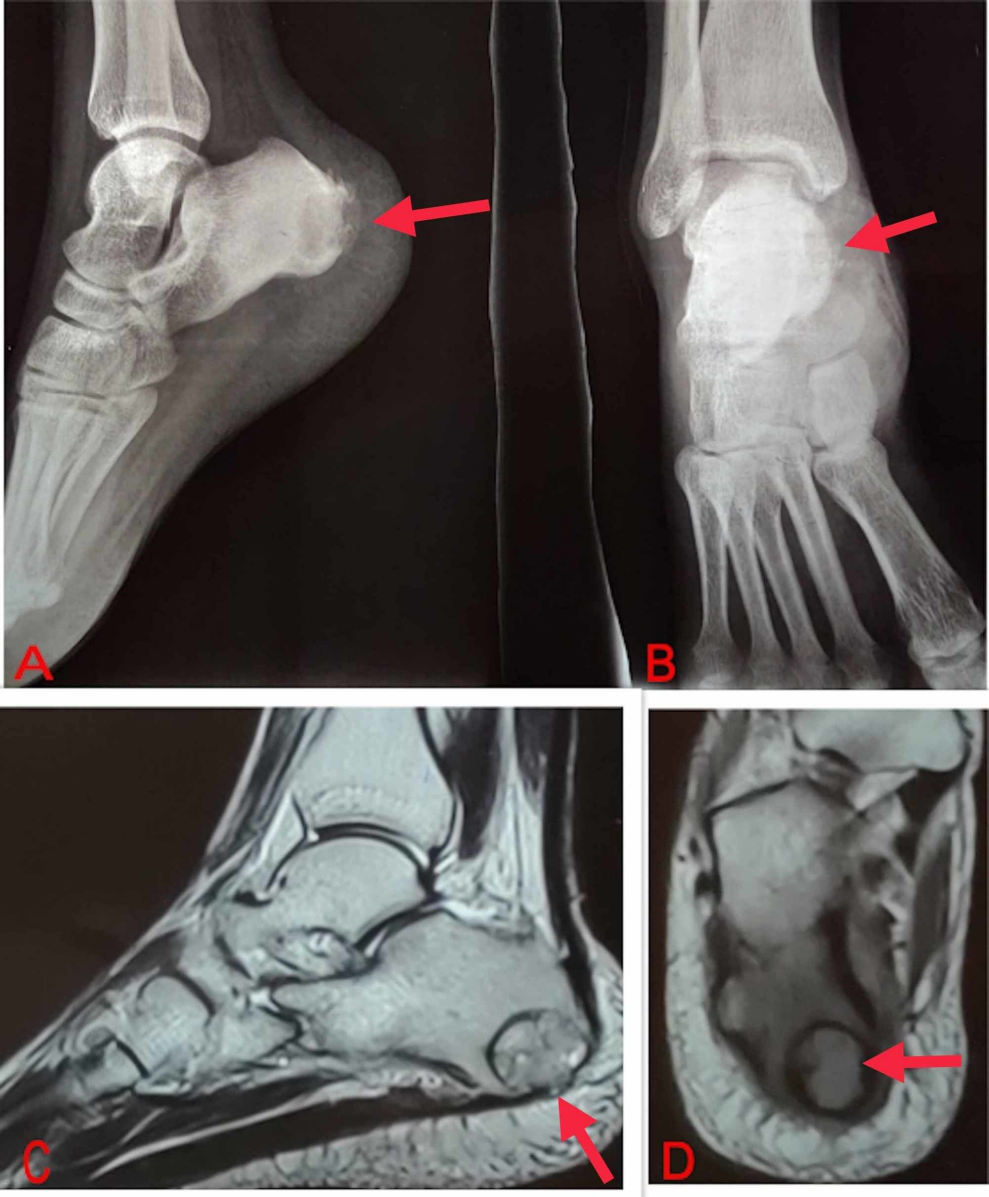
Preoperative radiographic assessment of the lesion. An expansile, eccentric, lytic lesion with non-sclerotic rim in the right calcaneus on X- rays (A, B); a well-defined hyperintense lesion with cystic areas abutting the posterior and medial surfaces of the calcaneus with hypointense margins and narrow zone of transition on MRI (C, D).

X-rays showed an expansile, lytic lesion of the calcaneus, and MRI showed a well-defined lesion with narrow zone of transition and no extraosseus spread (Figure 1). All biochemical investigations were normal. A core biopsy of the lesion was done. Samples sent for histopathology demonstrated characteristic multi-nucleated giant cells in a background of mononuclear stromal cells, features suggestive of a GCT.

**Figure 2:**
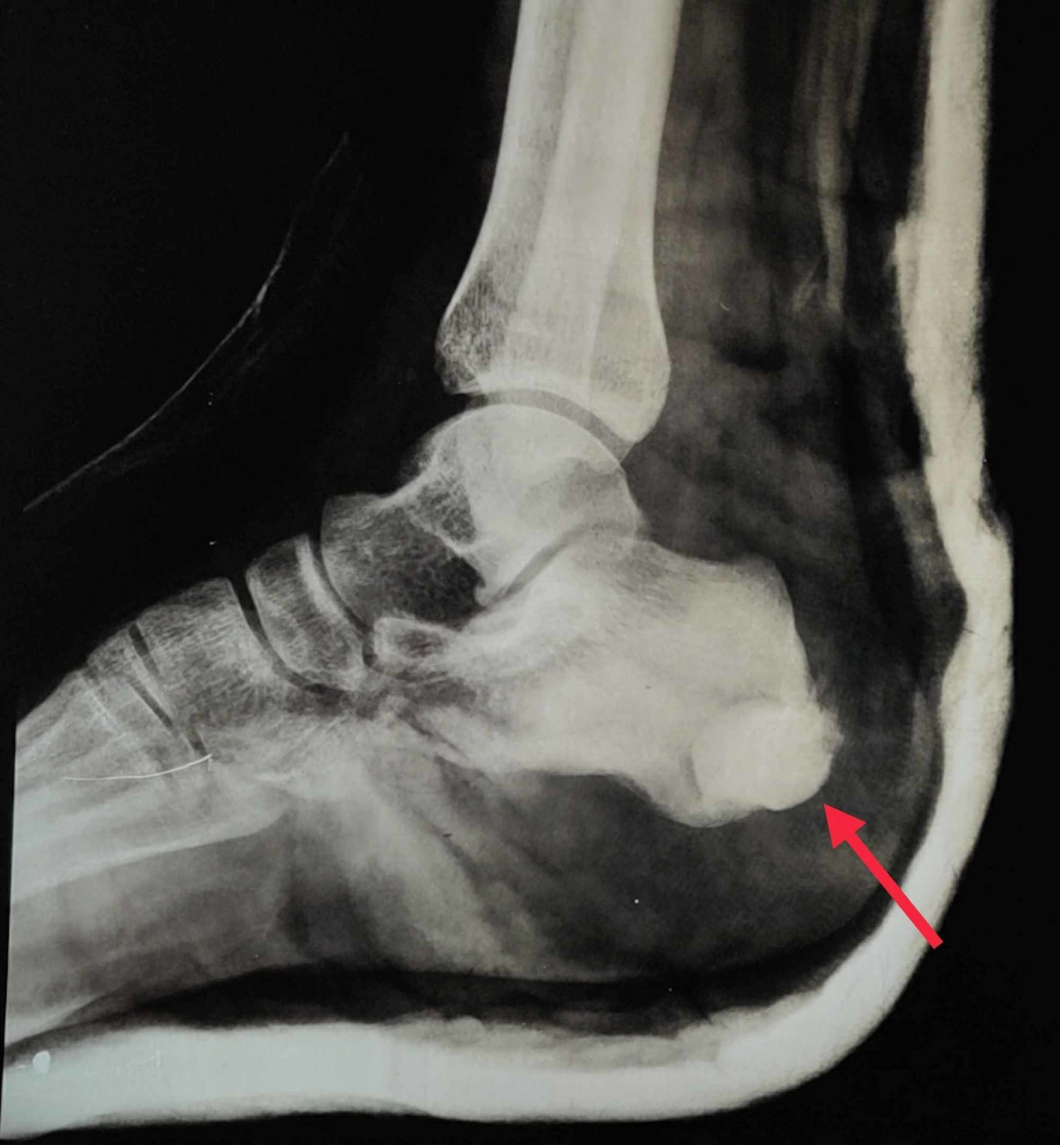
Curettage of the lesion and bone cementation.

After taking an informed consent, the patient was taken to the operating room. We used the medial approach to the calcaneus as the tumour was medially located. Curettage of the lesion was done with a mechanical burr and the defect was filled up with bone cement (Figure 2). Intraoperatively, the lesion was soft, greyish and well defined. The postoperative period was uneventful and the patient was discharged on the seventh day. The patient was put on a below-knee plaster slab for six weeks. Gradual weight bearing was started at six weeks.

**Figure 3:**
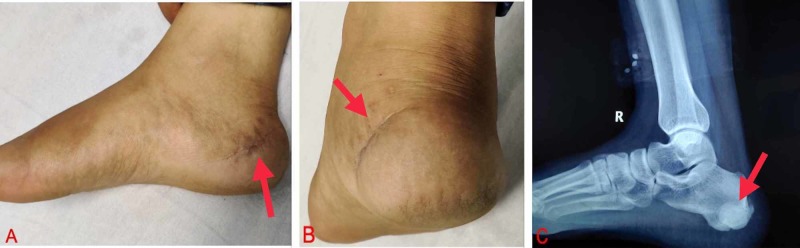
Clinical and radiographic assessment at two-year follow-up. Well-healed surgical scar with quiescent local site (A, B); X-ray having no evidence of recurrence at two-year follow-up (C).

The patient was followed up every three months for a year, and then six-monthly. At two-year follow-up, the patient was ambulatory and symptom-free and radiographs showed no evidence of recurrence (Figure 3).

## Discussion

The foot is an uncommon location for osseous tumours, comprising 3% of all skeletal tumours of which about one-third reside in the calcaneus [5]. GCT constitutes 1.2% of calcaneal tumours making it a rare entity [3]. A patient with GCT of the calcaneus generally presents in the age group of 30-40 years with heel pain and swelling. However, the age of our patient at the time of diagnosis was 17 years. So, our case highlights the fact that GCT can occur at unusual locations and in unusual age groups. It also asserts the importance of the surgeon's level of awareness and adequate preoperative workup, including biopsy, before proceeding to definitive treatment in such cases.

A typical GCT is an expansile, lytic lesion with well-defined, non-sclerotic margins and eccentric location [6]. On gross pathology, GCT is a soft friable dark tissue with associated areas of cystic and necrotic changes. Histology shows mononuclear stromal cells and characteristic multinucleated giant cells [7].

Considering the local aggressiveness of GCT, early detection and intervention is required. Treatment of calcaneal GCT is surgical. Curettage and bone grafting or placement of bone cement is the first line of management. However, addition of mechanical burr drilling of the tumour or cryoablation is now recommended considering the high rates of recurrence [8]. Most cases with local recurrence present within three years of primary surgery. Our patient, although symptom-free at two-year follow-up, should be monitored regularly not only for recurrence but also due to a 1%-9% risk of malignant transformation [9].

## Conclusions

GCT of the calcaneus is a rare entity and presents with pain and swelling in the heel. Although generally seen in the fourth decade of life, it can present in unusual age groups, as seen in our case. In addition a high level of awareness of the surgeon, adequate preoperative workup, including biopsy, is required to pick unusual tumours in unusual locations GCT of the calcaneus is associated with high recurrence rates and potentially aggressive behaviour. Detection and intervention in early stages can prevent local spread of the tumour and thus, avoid radical procedures like calcanectomy and amputation. Close follow-up is required to detect recurrence or metastasis, if any, at an early stage.
